# Early effects of LT3 + LT4 combination therapy on quality of life in hypothyroid patients: a randomized, double-blind, parallel-group comparison trial

**DOI:** 10.1186/s12902-025-01840-4

**Published:** 2025-01-26

**Authors:** Fatemeh Hajtalebi, Fariba Alaei-Shahmiri, Fatemeh Golgiri, Najmeh Shahini, Hamideh Akbari, Kasra Assadian, Seyedarad Mosalamiaghili

**Affiliations:** 1 Clinical Research Development Unit (CRDU), Sayad Shirazi Hospital, Golestan University of Medical Sciences, Gorgan, Iran; 2 Endocrine Research Center, Institute of Endocrinology and Metabolism, Iran University of Medical Sciences (IUMS), Tehran, Iran; 3 Clinical Research Development Unit (CRDU), Agh ghala Hospital, Golestan University of Medical Sciences, Gorgan, Iran; 4 Erdington & Kingstanding CMHT, Birmangham and Solihull Mental Health NHS Foundation Trust, Birmangham, UK; 5 Student Research Committee, Shiraz University of Medical Sciences, Shiraz, Iran; 6 Golestan Rheumatology Research Center, Golestan University of Medical Sciences, Gorgan, Iran

**Keywords:** Hypothyroidism, Levothyroxine, Triiodothyronine, Combination therapy, Quality of life

## Abstract

**Background:**

This study aimed to evaluate the impact of combined levothyroxine (LT4) and triiodothyronine (LT3) therapy on quality of life in patients with primary hypothyroidism.

**Methods:**

In a randomized, double-blind, parallel-group trial, 151 Iranian patients diagnosed with primary hypothyroidism between 2020 and 2021 were enrolled. One group received LT4 alone (*n* = 80), while the other received LT4 and LT3 (*n* = 71) for a minimum of six months. The primary outcome was quality of life assessed using the SF-36V1 questionnaire, and the secondary endpoints included clinical and laboratory measurements.

**Results:**

In the LT4 + LT3 group, a significant reduction in TSH levels (*p* < 0.05) was observed compared to baseline. While no significant differences emerged between the groups in terms of blood pressure, lipid profiles (except for low-density lipoprotein cholesterol), or body weight, there were notable improvements in physical functioning and bodily pain in the LT4 + LT3 group compared to the LT4 + placebo group. Compared with baseline, combination therapy significantly increased the physical component summary score after six months, but the difference was not significant.

**Conclusion:**

Combination therapy may benefit patients with primary hypothyroidism, particularly those experiencing body pain or physical function issues. However, the overall impact on quality of life remains inconclusive, as evidenced by the scores for the mental component. Further research is needed to determine the broader implications of this therapy. This study provides valuable insights into the potential advantages of combining LT4 and LT3 in the management of primary hypothyroidism.

**Trial registration:**

The study was registered with the Iranian Registry of Clinical Trials (IRCT) and assigned the registration number IRCT20200410047012N1 on 2022–08-07.

Trial registration number: IRCT20200410047012N1.

Date of registration: 2020–06-12.

## Background

Levothyroxine therapy (LT4) is a standard treatment for hypothyroidism, a prevalent endocrine disease. This medication has a half-life of 6.2 and 7.5 days in euthyroid and hypothyroid patients, respectively, and upon daily oral administration of LT4, thyroxine (T4) blood levels reach a state of stability [1, 2]. Nevertheless, despite achieving normal thyroid-stimulating hormone (TSH) levels, approximately 5 to 15% of patients report dissatisfaction with their treatment and continue to experience a significant decline in quality of life [3–6]. The exact reason for this phenomenon remains unknown but may be attributed to abnormally low triiodothyronine (T3) secretion by the thyroid or insufficient peripheral T3 production [1, 7]. Consequently, there have been proposals for combination therapy involving levothyroxine/liothyronine (LT4/LT3) [8].

The concept of LT4 + LT3 combination therapy has been suggested since the early twentieth century; however, its impact on patients' quality of life has remained a topic of ongoing debate [7]. Nygaard et al. [9] conducted a double-blind crossover randomized controlled trial (RCT) to assess the effect of LT4 + LT3 (versus LT4 alone) on quality of life. Their findings indicated a significant difference in the general health and vitality domains, though not in the social functioning and mental health domains. Conversely, over the past two decades, several studies have reported that LT4 + LT3 combination therapy did not enhance patients' quality of life [7, 10–12].

Due to conflicting evidence in the available literature, the National Institute for Health and Care Excellence has identified this subject as a high-priority research area in the management of hypothyroidism [13]. These authors emphasized the urgent need for high-quality RCTs to examine the efficacy and cost-effectiveness of T4–T3 combination treatment in individuals with hypothyroidism who do not respond to levothyroxine monotherapy.

In this context, it should also be noted that most studies on LT4 + LT3 combination therapy have been conducted in Western European and American populations, raising questions about whether this combination therapy is superior to LT4 monotherapy, especially among Asian populations [7]. As a result, the current RCT was undertaken to evaluate the impact of combined LT4 + LT3 therapy (compared to LT4 therapy alone) on quality of life in a Middle Eastern population.

## Materials and methods

### Trial design

We conducted a randomized, double-blind, parallel-group comparison trial at a 1:1 ratio, which took place at the outpatient clinic of an academic medical center in Gorgan, Iran. The study consisted of two phases: the baseline (April–October 2020) and the 6-month follow-up (October 2020-April 2021). Treatment was started immediately after randomization and continued for six months. Participants underwent evaluations at baseline (before treatment) and six months after treatment initiation, including clinical interviews and clinical testing. This randomized controlled trial was conducted and reported in accordance with the Consolidated Standards of Reporting Trials (CONSORT) guidelines [14].

### Participant selection

We included all eligible patients referred to the university hospital endocrine clinic and met our predefined criteria for inclusion and exclusion. The inclusion criteria necessitated that participants be older than 16 years of age; possess proficiency in reading and understanding the Persian language; have a confirmed diagnosis of overt hypothyroidism established at least six months before inclusion; maintain a stable and consistent regimen of LT4 monotherapy for a minimum of three months before inclusion; and self-report signs and symptoms of hypothyroidism, including fatigue, mood changes, weight gain, lethargy, decreased psychomotor performance, cognitive issues, depression, and disturbances, despite having normal thyroid hormone levels. The exclusion criteria included individuals who were pregnant or planning pregnancy within the subsequent six months; who had a history of drug or alcohol addiction; who had preexisting malignancy, CVD, renal or chronic liver disease, depression, anxiety, or any mental illness; who used psychiatric medication for at least six months before inclusion; and who had postsurgical hypothyroidism or subclinical hypothyroidism. Patients with hypothyroidism following radioiodine therapy were excluded from the study. Additionally, individuals on amiodarone therapy were also excluded due to its potential effects on thyroid function. None of the participants were receiving selenium supplements or statins at the time of the study, ensuring that these factors did not confound the results related to thyroid function and lipid profiles.

### Randomization

Patients in this study were randomly assigned to either the LT4 + placebo group or the LT4 + LT3 treatment group. An independent, trained researcher generated a list of random allocation cards using computer-generated random numbers. To prevent confusion, the researcher maintained the original random allocation sequences in inaccessible locations and worked with a copy.

### Interventions

Patients were randomized to either continue their usual LT4 dose (initially averaging 100 µg/day) in combination with a placebo (to ensure blinding) for six months (Group 1) or transition to combination therapy of LT4 (LT4 dose at the time of inclusion—50 µg) and LT3 (6.25 μg twice daily, administered in the morning and afternoon) (Group 2). The reduced LT4 dose was chosen because 100 μg of LT4 tablets are available in our country, preventing standard 14:1 T4:T3 ratio dosing from being feasible. The placebo was "liothyronine," which was labeled "approved for research purposes only." The placebo was placed into envelopes based on the allocation orders by another independent nurse who was unaware of the study. Patient IDs, visit dates, and other relevant information were recorded in each envelope. Neither the patients nor the evaluating physicians were aware of the treatment. After six months, another independent researcher not informed about the therapy assessed the patients' health-related quality of life scores.

### Clinical and laboratory measurements

The weight and height of the study participants were recorded with their shoes removed and while they wore lightweight clothing. Weight was taken to the nearest 100 g, and height was measured with participants standing upright using tape, ensuring their shoulders were aligned. Body mass index (BMI) was calculated as the weight in kilograms divided by the square of the height in meters (kg/m^2^). Systolic and diastolic blood pressure (SBP and DBP) were measured from the right arm following a 15-min rest in a seated position. All blood samples were collected in the morning, approximately 1–2 h after medication administration. Serum thyroid-stimulating hormone (TSH) levels were assessed using an immunocytometric assay (LIAISON TSH, Byk Gulden Italia, Milan, Italy). Total cholesterol (TC), high-density lipoprotein cholesterol (HDL-C), low-density lipoprotein cholesterol (LDL-C), and triglyceride (TG) levels were determined through enzymatic colorimetric methods (Pars Azmoon, Iran). Due to limitations, we could not assess triiodothyronine (T3) or T4 levels. These assessments were conducted at baseline and during follow-up using the same standardized approach. Physical activity levels were defined using the International Physical Activity Questionnaire short form (IPAQ20). This questionnaire comprises seven questions designed to yield domain-specific scores for walking, moderate-intensity, and vigorous-intensity physical activity, reflecting participants' activity levels during the seven days preceding the interview [15].

### Assessment of quality of life

Patients self-administered the 36-item Short-Form Health Survey version 1 (SF-36V1) questionnaire [16] at both baseline and follow-up. The SF-36V1 (0–100) was selected as the generic quality-of-life instrument and was previously validated in the Iranian population [17]. This questionnaire has been utilized in assessing patients with hypothyroidism [11, 12]. In its entirety, the short form comprises 36 questions encompassing eight health-related domains: physical functioning (10 items), limitations in physical activities due to health issues (4 items), bodily pain (2 items), general health perceptions (5 items), vitality (measuring energy levels and fatigue) (4 items), social functioning (2 items), role limitations due to emotional problems (3 items), and overall mental health (assessing psychological distress and well-being) (5 items). Additionally, it includes the calculation of physical and mental component summary scores derived from these eight health dimensions using a proprietary algorithm [18].

### Endpoints

The primary aim of this study was to examine changes in physical component summary (PCS) and mental component summary (MCS) scores within the LT4 + LT3 treatment group compared to those in participants who received LT4 + placebo.

Furthermore, the secondary outcomes of this study included changes in eight health-related domains and clinical variables, including weight, SBP, DBP, TC, TG, LDL-C, HDL-C, TSH, and physical activity.

The sample size was calculated using the following formula according to the primary outcomes [19]: ${n=\frac{{2({Z}_{1-\frac{\alpha }{2}}+{Z}_{1-\beta })}^{2}\delta }{{d}^{2}}}^{2}$

Considering a 5% margin of error and 90% statistical power and accounting for a 10% potential loss to follow-up or withdrawal, we determined that a sample size of 158 participants for both the LT4 + placebo and LT3 + LT4 groups would be sufficient to detect a 2-point difference in the physical or mental component summary scores of the SF-36V1 questionnaire [9, 11, 12]. As reported by Walsh et al. [11], a 2-point difference in the SF-36V1 physical or mental component summary score has been deemed clinically meaningful among individuals with and without thyroid disease. Additionally, we conducted a power analysis to assess the statistical power of the mean difference across all eight categories of the SF-36V1 in our study, and the minimum power exceeded 90%.

### Statistical analysis

For the statistical analysis, the baseline characteristics of the study population are presented as the mean ± standard deviation (SD) for continuous variables and as the frequency (%) for categorical variables. To compare these baseline characteristics between treatment groups, the t-test and the chi-square test were employed for continuous and categorical variables, respectively. For within-group comparisons between baseline and follow-up visits for continuous variables, paired t-tests or Wilcoxon signed-rank tests were used. Furthermore, to assess differences between treatment groups (between-group comparisons), the independent t-test or Mann‒Whitney U test was employed as appropriate. p values ≤ 0.05 were considered to indicate statistical significance. All the statistical analyses were conducted using SPSS version 20 statistical software.

### Ethics

The study received approval from the Ethics Committee of Gorgan University of Medical Sciences under the reference IR.GOUMS.REC.1399.016 (https://ethics.research.ac.ir/EthicsProposalViewEn.php?id=128312). Additionally, written informed consent was acquired from all participating patients. Furthermore, the study was registered with the Iranian Registry of Clinical Trials (IRCT) under registration number IRCT20200410047012N1, and the detailed information is available at https://www.irct.ir/trial/47402.

## Results

**Fig. 1:**
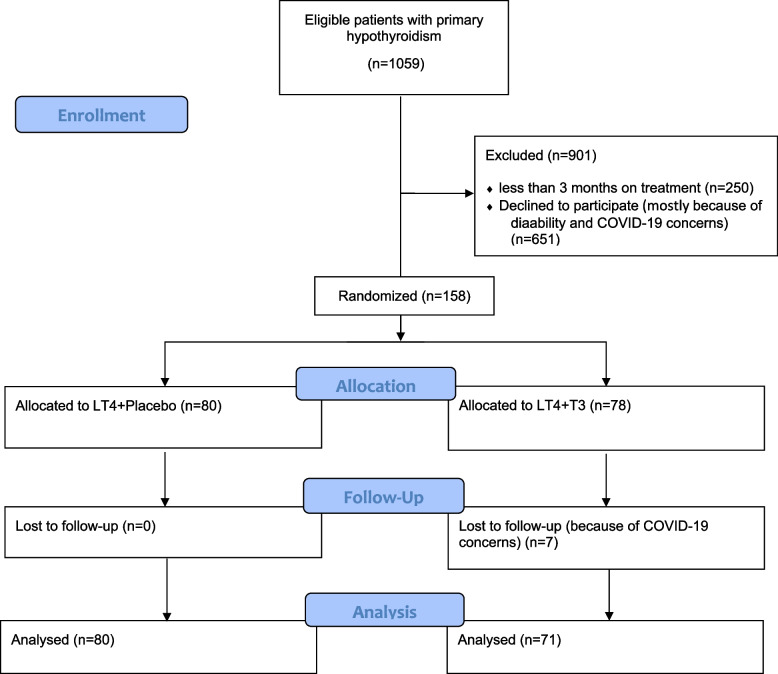
Flow chart of patients in the study

Of the 158 initially randomized patients, 151 (95.6%) completed the study, while seven patients from the LT4 + LT3 group voluntarily withdrew due to COVID-19 concerns. These seven individuals did not contribute any data at baseline or the six-month follow-up and were consequently excluded from the analysis (Fig. 1).

The baseline characteristics of the patients in the treatment groups are detailed in Table 1. The study population comprised 71 patients in the LT4 + LT3 subgroup and 80 in the LT4 + placebo subgroup. The participants in the LT4 + LT3 subgroup had a mean age ± SD of 42.24 ± 10.17 years, while those in the LT4 + placebo subgroup had a mean age of 43.08 ± 11.36 years. Generally, the two treatment groups exhibited comparable baseline characteristics, except for the mean DBP, which was significantly greater in the LT4 + LT3 group.

**Table 1:** Baseline characteristics of the patients by treatment groups

	**LT4 + Placebo** **(*N* = 80)**	**LT4 + LT3** **(*N* = 71)**
Age, year	43.08 ± 11.36	42.24 ± 10.17
Gender, female	74 (92.5%)	70 (98.6%)
Weight, kg	72.10 ± 14.0	75.85 ± 12.8
SBP, mmHg	114.4 ± 14.4	114.8 ± 13.8
DBP, mmHg	71.75 ± 10.0	75.26 ± 7.8
TSH, mU/L	2(1.4–2.9)	2.4(1.4–3.3)
Physical activity, Met-min/week	132.0(33.0–240.0)	89.0(33.5–175.5)

Values are mean ± SD for normally distributed, median (Q1-Q3) for skewed continuous variables, and n (%) for categorical variables were reported

*SBP* systolic blood pressure, *DBP* diastolic blood pressure, *TSH* thyroid-stimulating hormone

Clinical and biochemical data of patients in both groups were assessed before and after the intervention, as outlined in Table 2. In both treatment groups, serum LDL-C levels were significantly lower after six months compared to baseline [median (IQR); LT4 + placebo group: 93.5 (78–108.5) vs. 90.0 (70.0–101.5) mg/dL, p value = 0.02; LT4 + LT3 group: 95.0 (75.5–115.0) vs. 85.0 (75.0–102.0) mg/dL, p value = 0.007; between-group p value = 0.57]. The findings also revealed a significant reduction in TSH levels after six months in patients in the LT4 + LT3 subgroup [median (IQR): 2.4 (1.4–3.3) vs. 1.80 (1.10–3.20) mU/L, p value = 0.02]. Additionally, within the LT4 + placebo group, there was a decrease in physical activity over the 6-month period. No significant changes were observed after six months relative to baseline in terms of weight, SBP, DBP, TG, TC, or HDL-C. Furthermore, considering between-group comparisons, the differences in changes from baseline values of clinical and biochemical variables between the LT4 + placebo and LT4 + LT3 groups did not reach statistical significance.

**Table 2:** Comparison in changes of clinical and biochemical variables at baseline and 6 months

	**LT4 + Placebo**			**LT4 + LT3**			**Between-group comparison**	
	**Baseline**	**6 months**	***p*-value†**	**Baseline**	**6 months**	***p*-value†**	**Difference** **(95% CI)**^**a**^	*p*-value*
Weight, kg	72.10 ± 14.0	72.10 ± 13.91	1.0	75.85 ± 12.8	75.97 ± 12.26	0.65	0.13 (-0.51, 0.76)	0.69
SBP, mmHg	114.4 ± 14.4	113.63 ± 12.13	0.38	114.8 ± 13.8	114.64 ± 13.60	0.91	0.59 (-2.5, 3.7)	0.71
DBP, mmHg	71.7 ± 10.0	70.9 ± 8.0	0.39	75.3 ± 7.8	74.6 ± 7.2	0.53	0.12 (-2.71, 2.95)	0.93
TSH, mU/L	2(1.4–2.9)	2(1.31–2.67)	0.06	2.4(1.4–3.3)	1.80(1.10–3.20)	**0.02**	0.14 (-0.12, 0.41)	0.29
Physical activity, Met-min/week	132.0(33.0–240.0)	132.0(33.0–237.0)	**0.02**	89.0(33.0–175.0)	90.0 (33.0–183.0)	0.11	0.0 (0.0–0.0)	0.96

Values are mean ± SD for normally distributed, median (Q1-Q3) for skewed continuous variables, and n (%) for categorical variables were reported

*SBP* systolic blood pressure, *DBP* diastolic blood pressure, *TSH* thyroid-stimulating hormone, *CI* confidence interval

^a^Mean and median difference between treatment groups, as appropriate

^†^ *p*-values compare the changes from the baseline within each treatment group using paired t-test or Wilcoxon signed-rank test as appropriate

^*^ *p*-values compare the changes from the baseline between two treatment groups using an independent t-test or Mann–Whitney U Test as appropriate

The results of the impact of the LT4 + LT3 regimen versus the LT4 + placebo regimen on physical and psychosocial outcomes are displayed in Tables 3 and 4. Among the eight subscales of the SF-36V1, physical functioning (p value = 0.04) and bodily pain (p value = 0.004) significantly differed between the LT4 + LT3 and LT4 + placebo groups. In the combined LT4 + LT3 group, the median (IQR) physical function score increased from 65.0 (45.0–85.0) to 80.0 (60.0–90.0) after six months, while the corresponding value for the LT3 + placebo group was 75.0 (55.0–90.0) at baseline and 77.5 (56.25–90.0) at the 6-month follow-up. Additionally, the median (IQR) scores for psychological distress and well-being (55.7 ± 18.1 vs. 59.3 ± 18.2 from baseline to 6 months, respectively; *p* = 0.04), as well as for general health perceptions (54.4 ± 17.2 vs. 58.1 ± 16.5 from baseline to 6 months, respectively; *p* = 0.04), significantly increased after six months in the combined LT3 + LT4 treatment group, although no such changes were observed in the LT4 + placebo group. However, between-group differences did not reach statistical significance. A similar pattern was also noted for the physical component summary score, with the combined LT3 + LT4 treatment group (baseline: 42.79 ± 8.68 vs. after six months: 45.49 ± 9.03; *p* = 0.003) displaying an increase compared to the LT4 + placebo group (baseline: 44.49 ± 10.09 vs. after six months: 45.39 ± 9.49, *p* = 0.08; between-group *p* = 0.07). No significant changes in the mean values of the mental component summary score were observed in either the LT3 + LT4 or LT4 + placebo groups.

**Table 3:** Changes in SF-36V1 scores at baseline and 6 months

	**LT4 + Placebo**			**LT4 + LT3**		
	**Baseline**	**6 months**	***p*-value**	**Baseline**	**6 months**	***p*-value**
Physical functioning	75.0 (55.0–90.0)	77.5 (56.25–90.0)	**0.01**	65.0 (45.0–85.0)	80.0 (60.0–90.0)	**0.002**
Social functioning	61.56 ± 23.09	60.62 ± 21.33	0.38	60.21 ± 21.57	59.86 ± 20.80	0.88
Vitality (energy and fatigue)	55.50 ± 20.24	54.56 ± 20.22	0.48	52.46 ± 19.75	54.86 ± 18.28	0.21
General mental health	60.40 ± 18.85	60.30 ± 18.86	0.94	55.71 ± 18.13	59.32 ± 18.21	**0.04**
Bodily pain	67.5 (45.0–80.0)	65.0 (45.0–83.75)	0.8	55.0 (32.5–77.5)	57.5 (45.0–78.7)	0.09
Limitations in physical activities	75.0 (25.0–100.0)	75.0 (12.5–100.0)	0.23	50.0 (25.0–100.0)	75.0 (25.0–100.0)	0.27
General health perceptions	56.09 ± 20.64	56.95 ± 19.31	0.39	54.40 ± 17.17	58.09 ± 16.54	**0.04**
Limitations due to emotional problems	53.75 ± 44.84	55.42 ± 42.74	0.59	49.29 ± 42.11	53.52 ± 44.89	0.38
PCS score	44.49 ± 10.09	45.39 ± 9.49	0.08	42.79 ± 8.68	45.50 ± 9.04	**0.003**
MCS score	42.24 ± 10.56	41.75 ± 9.94	0.45	40.96 ± 10.63	41.32 ± 9.90	0.71

Values are mean ± SD for normally distributed, median (Q1-Q3) for skewed continuous variables, and n (%) for categorical variables were reported. *CI* confidence interval, *General mental health* psychological distress and well-being, *PCS* Physical component summary, *MCS* Mental component summary; *p*-values compare the changes from baseline within each treatment group using paired t-test or Wilcoxon signed-rank test as appropriate

**Table 4:** Comparing SF-36V1 scores between the study groups

SF-36V1 scores	**Between-group difference** **(95% CI)**^**a**^	*p*-value*
Physical functioning	-5.0 (-10.0, 0.0)	**0.04**
Social functioning	0.58(-4.60, 5.80)	0.82
Vitality (energy and fatigue)	0.0 (-5.0, 0.0)	0.26
General mental health	-4.0 (-0.4, 0.0)	0.19
Bodily pain	-7.50 (-10.0, 0.0)	**0.004**
Limitations in physical activities	0.0 (0.0, 0.0)	0.22
General health perceptions	0.0 (-6.25, 0.0)	0.07
Limitations due to emotional problems	0.0 (0.0, 0.0)	0.33
PCS score	-1.44 (-3.6, 0.11)	0.07
MCS score	0.18 (-1.81, 1.75)	0.84

^a^Mean or median difference between treatment groups, as appropriate; *CI* confidence interval, *General mental health* psychological distress and well-being; *PCS* Physical component summary, *MCS* Mental component summary; *p-values compare the changes from baseline between two treatment groups using independent t-test or Mann–Whitney U Test as appropriate

## Discussion

We conducted a randomized, double-blind, parallel-group comparison trial to assess whether LT4 + LT3 enhances clinical, biomedical, and quality-of-life outcomes in hypothyroid patients. Our results indicated significant improvements in psychological distress, general well-being, general health perceptions, body pain, and physical activity in the LT4 + LT3 group compared to LT4 alone. Specifically, improvements were seen in physical functioning and bodily pain, which are important quality-of-life indicators. However, our data do not suggest any significant difference in the changes in body weight, blood pressure, serum lipids, and serum TSH between the two treatment groups.

Consistent with our findings, Michaelsson et al. observed significant improvements in quality of life among 23 patients who transitioned from LT4 to LT4 + LT3 therapy, as measured by the Thyroid Patient-Reported Outcome (ThyPRO-39) scale, during 3- and 12-month follow-up periods [20]. Similarly, in a study conducted by Bunevičius et al. involving 33 patients with hypothyroidism, individuals receiving LT4 + LT3 therapy exhibited improved neuropsychological function and mood compared to those receiving LT3 therapy [21].

Recent evidence suggests that LT4 monotherapy may not adequately restore tissue-specific levels of T3 due to the suppression of type 2 deiodinase (D2), which is critical for converting T4 to active T3 in peripheral tissues. This suppression can result in a state of "low tissue T3," particularly in D2-expressing tissues such as the brain, despite achieving normal serum TSH levels. The addition of LT3 aims to directly address this imbalance, potentially normalizing tissue T3 levels and alleviating persistent symptoms like fatigue, brain fog, and mood disturbances​ [22, 23]. Furthermore, studies have shown that LT3 + LT4 therapy can modulate the fT3/fT4 ratio more effectively than LT4 alone, approximating the physiological secretion ratio of thyroid hormones. This adjustment may enhance thyroid hormone signaling at the cellular level, contributing to improved metabolic and psychological outcomes in a subset of patients​ [24].

However, there are studies with contrasting results. Valizadeh et al. revealed that combined treatment with LT4 + LT3, compared to LT4 alone, did not improve psychosocial outcomes in hypothyroid Iranian patients. They used a randomized crossover design, with 30 patients in the LT4 + LT3 treatment group and 30 in the LT4 alone group. The General Health Questionnaire-28 (GHQ-28) was utilized to assess the mental health status of patients in these two treatment groups after four months of follow-up. Additionally, they reported no enhancement in the metabolic rate of patients receiving LT4 + LT3 treatment compared to patients receiving monotherapy [25]. In another study utilizing a randomized parallel design and the Health-Related Quality of Life (HRQL) questionnaire, Clyde et al. demonstrated that, when compared to LT4 alone, the combination therapy of LT4 and LT3 did not result in improvements in quality of life, weight, SBP/DBP, or lipid profiles after four months [26]. A meta-analysis of eleven published articles from 1999 to 2005 also found no significant differences in the effectiveness of combination LT4 + LT3 versus LT4 therapy in bodily pain (4 studies), body weight (4 studies), TC (8 studies), TG (5 studies), LDL-C (4 studies), HDL-C (3 studies), depression (11 studies), anxiety (7 studies), and quality of life (7 studies) [27]. In another study conducted by Walsh et al., none of the SF-36 components differed between combination therapy and monotherapy; however, in our study, we observed significant differences in two subdomains: physical functioning and bodily pain [11]. They also reported an increase in the level of TSH during combination therapy, which may be attributed to the short half-life of T3. It is likely that during combined LT4 + LT3 treatment, the serum TSH concentration increases slightly as the serum T3 concentration decreases, reaching its highest point 24 h after the latest dose of thyroid hormone. Like our findings, Nygaard et al. demonstrated that serum TSH levels did not differ between LT3 + LT4 therapy and therapy with LT4 alone [9].

In a meta-analysis comprising ten randomized, double-blind trials involving 646 patients, Chao et al. demonstrated that T4 alone exhibited significant benefits for both physical and mental component summaries. They also reported that T4 + T3 therapy did not yield any improvement in clinical, mental, or physical variables. Based on the evidence, they suggested that T4 alone is a superior treatment option to combination therapy, as it also alleviates bodily pain and enhances physical functioning. The notable difference between LT4 and LT4 + LT3 may be linked to the daily production of triiodothyronine, the primary source of which is (approximately 80%) deiodination of T4 in extrathyroidal tissues [28].

The disparities in findings between studies that reported a significant improvement in quality of life with combination therapy and those that did not may be attributed to variations in sample sizes, the utilization of diverse questionnaires, or differences in study designs.

The recent Consensus Document developed by ATA/BTA/ETA emphasized that previous RCTs comparing LT3/LT4 therapy to LT4 monotherapy enrolled hypothyroid patients without considering whether they had persistent symptoms or dissatisfaction [29]. They noted, "It is possible that those individuals most likely to benefit from combination therapy may not yet have been included in trials in sufficient numbers to provide adequate power for detecting a response." Furthermore, given the drawbacks of a crossover design, which includes potential carryover effects and challenges in managing drop-outs or conducting a sufficiently long RCT, all consensus members concurred that "A future combination therapy trial should incorporate a parallel design." As a result, we opted for a parallel randomized design with a six-month follow-up period instead of a crossover design to facilitate the detection of practice or placebo effects. Several crossover designs have reported significant practice effects associated with repeated testing after combined therapy [21, 30]. Clyde et al. demonstrated that with a parallel design, effective practice and placebo effects can be observed within a 4-month follow-up period [26].

While LT4 monotherapy normalizes TSH levels, some patients still experience symptoms, indicating that LT4 alone may not fully restore cerebral thyroid hormone levels. Genetic polymorphisms affecting deiodinase enzymes could contribute to suboptimal T3 levels in the brain, potentially explaining these persistent symptoms​ [24]. Other factors, such as TSH levels, duration of hypothyroidism, marital status, pain levels, employment status, diet, antithyroid peroxidase antibodies, age, and genetics, may also influence quality of life. However, further investigations are needed to determine the exact associations between these factors and quality of life in patients with hypothyroidism [7].

This study possesses several strengths, including the use of a parallel randomization design, a larger sample size that enhances the study's statistical power, an extended follow-up period, and the recruitment of patients specifically experiencing inadequate relief of their hypothyroid symptoms. Furthermore, to the best of our knowledge, this is the first study of its kind in the Middle East and North Africa region that utilized the SF-36 questionnaire. This study has certain limitations. First, blood sample collection occurred one to two hours after hormone therapy, potentially impacting the serum hormone levels due to absorption peaks. Second, baseline measurements were unavailable for all participants, as randomization occurred before these measurements. Third, this study focused on the early effects of LT3 + LT4 combination therapy on patients' quality of life, with follow-up conducted over six months. Longer-term effects, with follow-ups at 12 and 24 months, remain to be explored in future studies. Finally, the loss of follow-up happened only in the LT4 + T3 group.

## Conclusion

In conclusion, our findings indicate that combined therapy with LT3 + LT4, but not LT4 alone, significantly increased the physical component summary score among patients with primary hypothyroidism after six months. However, no significant difference was observed between LT4 + LT3 and LT4 + placebo. Although the summary scores for physical and mental health components do not support the hypothesis that combined therapy improves quality of life, combination therapy should be considered for hypothyroidism patients experiencing issues related to body pain and physical activity.

## Data Availability

The data that support the findings of this study are not openly available due to reasons of sensitivity and are available from the corresponding author upon reasonable request.

## Abbreviations

LT4: Levothyroxine therapy
T4: Thyroxine
TSH: Thyroid-stimulating hormone
T3: Triiodothyronine
LT4/LT3: Levothyroxine/Liothyronine
RCT: Randomized control trial
TG: Triglycerides
HDL-C: High-density lipoprotein cholesterol
LDL-C: Low-density lipoprotein cholesterol
TC: Total cholesterol
SD: Standard deviation
IQR: Interquartile range
